# Temporal Dynamics of Physiological Integration Intensity in *Zoysia japonica* Under Heterogeneous Stress of Cadmium or/and Phenanthrene

**DOI:** 10.3390/plants14081230

**Published:** 2025-04-17

**Authors:** Sunan Xu, Yichen Liu, Xuemei Li, Zhonglin Chen, Lihong Zhang, Yue Li

**Affiliations:** 1 College of Environment Science, Liaoning University, Shenyang 110036, China; xusunan@lnu.edu.cn (S.X.); lychennn@126.com (Y.L.); chenzhonglin@1969163.com (Z.C.); 2 College of Life Science, Shenyang Normal University, Shenyang 110034, China; lxmls132@163.com

**Keywords:** clonal plant, HMs, PAHs, physiological integration intensity, photosynthetic parameters

## Abstract

Heavy metals (HMs) or/and polycyclic aromatic hydrocarbons (PAHs) stress have significant adverse effects on the photosynthetic function and SPAD values of plants. Physiological integration is the typical feature of clonal plants, which can mitigate the adverse effects on ramets under heterogeneous stress. However, the sustainability of physiological integration between clones over prolonged stress durations, the dynamics of integration intensity and potential differences under various stress types remain unclear. This study examined the effects of three different heterogeneous stresses—cadmium (Cd), phenanthrene (Phe), and a combination of Cd and Phe (Cd + Phe) on the physiological integration of *Zoysia japonica* at different time points. The results indicate that physiological integration significantly enhances SPAD value, net photosynthetic rate (P_N_), stomatal conductance (Cond), intercellular CO₂ concentration (C_i_), transpiration rate (Tr), and water use efficiency (WUE). However, the physiological integration intensity diminishes with prolonged stress exposure. In addition, among different stress types, the initial integration intensity was highest under the highest toxicity conditions, it decreased most rapidly, resulting in the lowest integration intensity during the later stages of stress. To sum up, this study highlights the role of physiological integration in maintaining the photosynthetic function of clonal plants under heterogeneous stress and elucidates the temporal changes in integration intensity under different stress conditions.

## 1. Introduction

Physiological integration, a defining feature of clonal plants that sets them apart from non-clonal species [1]. Through physiological integration, energy, water, and nutrient transmission, as well as signal conduction, can occur between clonal plant ramets via connections such as rhizomes and stolons [2,3,4]. Current studies have predominantly examined the role of physiological integration in modulating phenotypic plasticity, physiological responses, and photosynthetic performance of clonal plants under various abiotic stress conditions, including nutrient availability, water stress, light intensity, and salinity [5,6,7,8]. The connected ramets of moso bamboo can improved nitrogen use efficiency by translocating and sharing nitrogen [9]. The interconnected rhizome of *Phyllostachys. edulis* can increase the activity of antioxidant enzymes in water-deficient ramets under heterogeneous water conditions [10]. In the case of negative correlation of light, rhizome connection of dwarf bamboo significantly increase photosynthetic rate, non-structural carbohydrate content of unshaded ramets [11]. Physiological integration enhanced enzymes activity of catalase and peroxidasein both stressed and unstressed ramets of Bermuda grass (*Cynodon dactylon* [L.] Pers.) [12]. Despite these insights, the effects of stress duration and intensity on physiological integration remain underexplored, warranting further investigation into whether physiological integration dynamics alter with prolonged stress exposure.

As a typical clonal plant, *Zoysia japonica* is connected by strong stolons and exhibits high adaptability to environmental heterogeneity [13]. Through our previous studies, it has been demonstrated that *Z*. *japonica* possesses significant physiological integration ability under conditions of heterogeneous water and nutrient availability [14,15]. Furthermore, its widespread use in urban landscapes—such as home lawns, football fields, and golf courses—highlights its importance in urban greening [16,17]. Investigating the effects of persistent heterogeneous stress on *Z*. *japonica* is, therefore, of critical importance for ensuring the safety and sustainability of urban green spaces [18].

Over the years, with the development of various industrial activities, especially metal smelting, the problem caused by heavy metals (HMs) pollution has attracted more and more attention [19]. Cd is a typical heavy metal that exhibits high solubility, bioavailability, and capacity for absorption and enrichment in ecosystems [20]. Its intake and accumulation pose significant threats to plant, animal, and human health [21]. Simultaneously, polycyclic aromatic hydrocarbons (PAHs), such as those released from vehicle exhaust and waste incineration, have become pervasive atmospheric pollutants in urban environments [22]. Phenanthrene (Phe) is a three-ring PAHs that accumulates in soil through rainfall and atmospheric deposition [23]. Due to these factors, co-contamination of urban soils by multiple HMs and PAHs is increasingly common, posing a major threat to urban green spaces and public health. Studies have demonstrated the detrimental effects of such co-contamination on plant health. Under the combined stress of Cd and lead (Pb), the biomass and antioxidant enzyme activities of *Salvia miltiorrhiza* significantly decreased [24]. The maize plants exhibited a significant decrease in height and fresh weight when exposed to varying levels of pyrene, Cu, and Cd combinations for 57 d [25]. Likewise, Bermuda grass (*Cynodon dactylon* (L.) Pers.) under PAH and Cd contamination displays diminished growth rates, chlorophyll content, root activity, and biomass, along with elevated malondialdehyde levels and electrolyte leakage [26]. Similarly, *Medicago sativa* grown in pyrene- and HM-contaminated soils showed significantly lower growth parameters, biomass, and leaf pigment content, with markedly higher reactive oxygen species activity, proline/polyphenol levels, and metallothionein protein content [27]. These findings highlight the sensitivity of plants to HMs and PAHs, underscoring the urgency of further research into mitigation strategies.

In our study, we investigated the effects of heterogeneous stress, induced by Cd and/or Phe, on the photosynthetic activity, SPAD values, and physiological integration characteristics of *Z. japonica*. Specifically, we examined the trends in physiological integration strength under prolonged heterogeneous stress conditions. This allows us to understand physiological integration in plants, from its characteristics to its changing patterns. Such insights are crucial for elucidating the mechanisms and patterns through which clonal plants leverage physiological integration to optimize their growth and adaptability.

## 2. Results

### 2.1. Photosynthetic Parameter and SPAD of Z. japonica Ramets Under Cd Heterogeneous Stress

**Figure 1:**
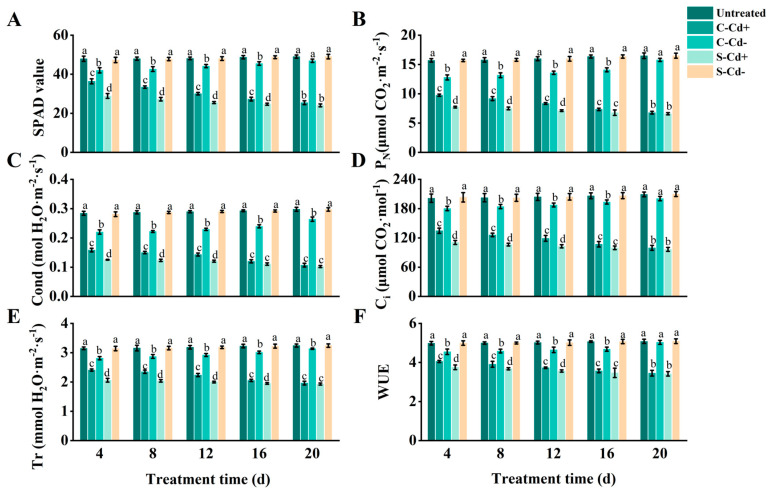
Photosynthetic parameters and SPAD values in connected and severed *Z. japonica* ramets under Cd heterogeneous stress at different times. SPAD value (**A**), P_N_ (**B**), Cond (**C**), C_i_ (**D**), Tr (**E**), and WUE (**F**). Values are mean ± SD for *n* = 9. Bars with different letters are statistically different at *p* < 0.05, as determined by the LSD test.

As shown in Figure 1, over the 20 d stress period, SPAD value, P_N_, Cond, C_i_, Tr, and WUE of the ramets under Cd heterogeneous stress (S-Cd+ and C-Cd+) were all significantly decreased compared to the untreated group, with the magnitude of reduction increasing over time. Notably, we also observed that the six indices (SPAD value, etc.) of C-Cd+ treatment were higher than the S-Cd+ treatment, although the rate of increase diminished as the stress duration extended. At 4 d, the six indices of C-Cd+ were 25.97%, 26.20%, 26.24%, 21.56%, 16.82%, and 7.96% higher than S-Cd+, respectively, with significant differences observed. However, at 20 d, the differences had narrowed to 5.63%, 2.66%, 4.78%, 3.22%, 1.79%, and 0.91%, respectively, and were no longer statistically significant. Additionally, the six indices for C-Cd- treatment were also lower than those of the untreated group, but the extent of reduction gradually decreased over time. Significant differences between C-Cd- and the untreated group were observed at 4, 8, 12, and 16 d. At 20 d, however, only Cond and Tr remained significantly different, while the other indices showed no significant differences. In contrast, the six indices for S-Cd- remained relatively stable and showed minimal changes compared to the untreated group throughout the 20 d stress period. During the 20 d experimental period, the S-Cd+ ramets exhibited leaf yellowing initiating from the margins. The leaves displayed a yellow-green coloration accompanied by brown spots and necrotic areas. Additionally, some leaves curled, crumpled, became smaller, and eventually wilted. The damage of C-Cd+ ramets was slightly less than that of S-Cd+; brown spots and necrotic areas were less, and there were almost no eventually wilted leaves. In contrast, the untreated and S-Cd- ramets grew with a creeping habit, featuring short internodes. Its leaves were dark green or bright green, with uniform coloration and a rich luster, demonstrating healthy growth without any signs of wilting. Due to physiological integration, C-Cd- ramets had basically no new leaf growth in the late stage of the experiment, and the edges of individual leaves turned yellow, and occasionally necrotic areas appeared.

### 2.2. Photosynthetic Parameter and SPAD of Z. japonica Ramets Under Phe Heterogeneous Stress

**Figure 2:**
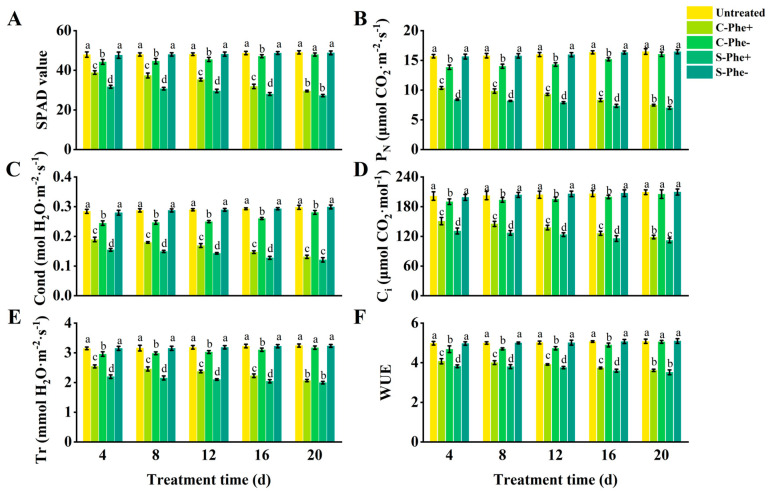
Photosynthetic parameters and SPAD value in connected and severed *Z. japonica* ramets under Phe heterogeneous stress at different times. SPAD value (**A**), P_N_ (**B**), Cond (**C**), C_i_ (**D**), Tr (**E**), and WUE (**F**). Values are mean ± SD for *n* = 9. Bars with different letters are statistically different at *p* < 0.05, as determined by the LSD test.

The SPAD value, P_N_, Cond, C_i_, Tr, and WUE of the ramets under Phe heterogeneous stress (S-Phe+ and C-Phe+) were gradually decreased with the duration of stress and showed significant differences compared to the untreated group (Figure 2). C-Phe+ ramets exhibited higher values for the six indices (SPAD value, etc.) compared to S-Phe+ ramets; however, the extent of this increase diminished over time. At 4 d, the six indices of C-Phe+ were 22.66%, 23.40%, 22.65%, 15.20%, 15.66%, and 6.69% higher than those of S-Phe+, respectively, with all differences being statistically significant. At 20 d, only 8.32%, 6.46%, 8.44%, 6.16%, 3.51%, and 2.84% were higher than S-Cd+, respectively, and there were no significant differences except for Cond. Meanwhile, C-Cd- ramets exhibited six lower indices compared to untreated ramets, but the reduction gradually decreased with prolonged stress duration. Significant differences between C-Cd- and untreated existed at 4, 8, 12, and 16 d. However, at 20 d, no significant differences remained except for C_i_. The six indices of S-Phe- also showed little change compared to the untreated group during the 20 d stress duration. During the 20 d experimental period, S-Phe+, C-Phe+, and C-Phe- ramets exhibited similar growth dynamics to those of Cd stress, but the degree of damage was obviously less than that of Cd stress. Untreated and S-Phe- ramets grew healthily, similar to Cd stress.

### 2.3. Photosynthetic Parameter and SPAD of Z. japonica Ramets Under Cd and Phe Heterogeneous Stress

**Figure 3:**
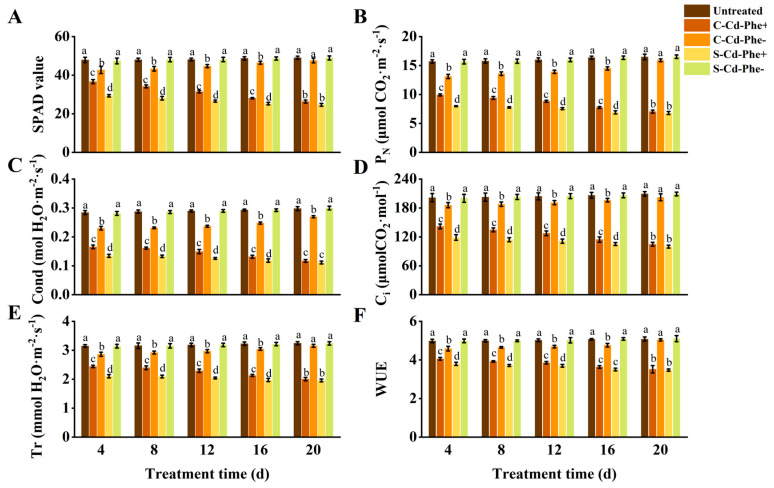
Photosynthetic parameters and SPAD value in connected and severed *Z. japonica* ramets under Cd and Phe heterogeneous stress at different times. SPAD value (**A**), P_N_ (**B**), Cond (**C**), C_i_ (**D**), Tr (**E**), and WUE (**F**). Values are mean ± SD for *n* = 9. Bars with different letters are statistically different at *p* < 0.05, as determined by the LSD test.

As depicted in Figure 3, the SPAD value, P_N_, Cond, C_i_, Tr, and WUE of Cd and Phe heterogeneous stress (S-Cd-Phe+ and C-Cd-Phe+) ramets significantly decreased compared to the untreated group. Specifically, connected treatment (C-Cd-Phe+) mitigated the decline in six indices (SPAD value, etc.) relative to severed treatment (S-Cd-Phe+). At 4 d, the six indices of C-Cd-Phe+ were 24.96%, 24.31%, 23.11%, 19.36%, 16.16%, and 6.97% higher than those of S-Cd-Phe+, respectively, and all showed significant differences. At 20 d, the increases between C-Cd-Phe+ and S-Cd-Phe+ had narrowed to 6.94%, 3.56%, 4.99%, 4.78%, 2.38%, and 1.29%, respectively, and none of these differences remained significant. Similar to the trends observed in C-Cd- and C-Phe- treatments, six indices of C-Cd-Phe- ramets significantly decreased compared to the untreated group at 4, 8, 12, and 16 d, but no significant differences were observed at 20 d. The six indices of S-Cd-Phe- showed minimal change compared to the untreated group throughout the entire stress duration either. During the 20 d experimental period, S-Cd-Phe+, C-Cd-Phe+, and C-Cd-Phe- ramets also exhibited similar growth dynamics to those of Cd stress and Phe stress; the degree of damage was less than that of Cd stress but more than that of Phe stress. Untreated and S-Phe- ramets grew healthily, similar to Cd stress and Phe stress.

### 2.4. Physiological Integration Intensity of Z. japonica Ramets Under Cd or/and Phe Heterogeneous Stress

**Figure 4:**
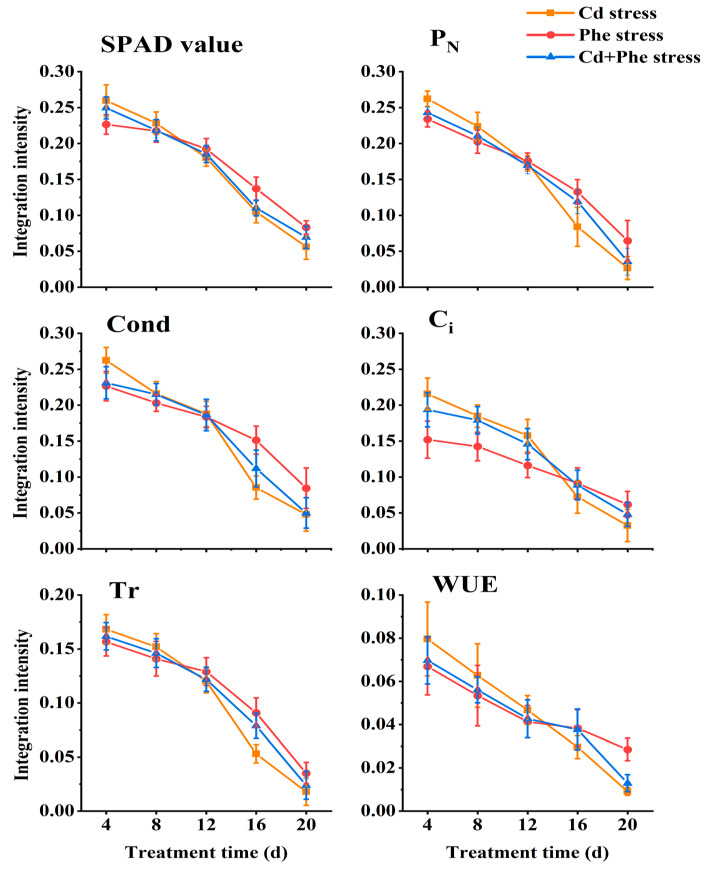
Physiological integration intensity of SPAD value, P_N_, Cond, C_i_, Tr, and WUE of *Z. japonica* ramets under Cd or/and Phe heterogeneous stress at different times. The values are calculated from the average of the relevant indicators.

Connectivity between ramets, different kinds of treatments (Cd, Phe, and Cd + Phe), duration, and their interaction had significant effects on all the photosynthetic parameters and SPAD values of *Z*. *japonica* ramets, except the effect of connectivity on C_i_ (Table 1). We employed Formulas 1–3 to quantify the physiological integration intensity of six parameters, including SPAD values, etc., across various treatments. Subsequently, we conducted comprehensive analyses to examine both the differences among treatments and the temporal trends observed during different durations. The physiological integration intensity of SPAD value, P_N_, Cond, C_i_, Tr, and WUE in *Z*. *japonica* ramets under Cd or/and Phe heterogeneous stress at different time points was shown in Figure 4. Across the three treatments, the physiological integration intensity of the six indices exhibited a declining trend with prolonged stress duration, but the rate of decrease varied significantly across treatments. At 4 d, physiological integration intensity of six indices (SPAD value, etc.) under Cd heterogeneous stress was the highest, followed by Cd-Phe stress, and Phe stress was the lowest. As the duration of heterogeneous stress increased, the physiological integration intensity of six indices under Cd, Phe, and Cd-Phe gradually decreased. Specifically, the physiological integration intensity of the other five indices (except C_i_) under Cd, Phe, and Cd-Phe heterogeneous stress became nearly identical at 12 d (C_i_ reached this point at 16 d). At 20 d, the physiological integration intensity of six indices under Cd heterogeneous stress had become the lowest, followed by Cd + Phe stress, while Phe stress exhibited the highest intensity. This trend contrasted sharply with the results at 4 d.

## 3. Discussion

Photosynthesis, as the fundamental physiological process in plants, serves as the primary source of material and energy for plant growth [28]. Chlorophyll plays a pivotal role in photosynthesis; maintaining a certain level of chlorophyll is essential for normal photosynthesis activity [29]. However, contamination by HMs and PAHs can disrupt the membrane systems of chloroplasts, mitochondria, and cystoids in plants, consequently affecting the chlorophyll content and inhibiting the photosynthetic process [27,30,31]. Di et al. [32] indicated that the photosynthetic characteristics of *Rhododendron simsii* were significantly reduced under Cd stress. Similarly, Hu et al. [33] found that higher Cd concentrations decreased the stomatal density in *Picris Divaricata vant* leaves, leading to stomatal limitations and impaired photosynthesis. Consistent with these findings, in our study, Cd or/and Phe stress significantly decreased the SPAD value, P_N_, Cond, C_i_, Tr, and WUE in *Z. japonica* clonal ramets. Additionally, numerous studies have established that pollutants, especially HMs, tend to accumulate progressively in plants as stress duration increases [34,35], ultimately causing damage to various physiological and metabolic processes. This accumulation phenomenon likely explains the continuous decline in SPAD values and photosynthetic parameters observed in our study with prolonged exposure to Cd and/or Phe heterogeneous stress.

Physiological integration represents a core adaptive strategy of clonal plants to mitigate abiotic stress, effectively reducing stress-induced damage [8,36,37]. In our study, under Cd or/and Phe heterogeneous stress, physiological integration also increased the SPAD value and photosynthetic parameters of the connected stressed ramets. This enhancement can be attributed to the directional resource transport and stress response signal regulation, consistent with previous findings on material transport and signal transduction in physiological integration by Zhang et al. and Duan et al. [38,39]. However, we observed a gradual attenuation of physiological integration effects, manifested by progressively smaller increases in SPAD values and photosynthetic parameters of recipient ramets (C-Cd+, C-Phe+, and C-Cd-Phe+ ramets) with prolonged stress exposure. This phenomenon can be explained by several key factors. a. HM accumulation: heavy metal ions were also transferred to the donor ramets (C-Cd-, C-Phe-, and C-Cd-Phe- ramets) during resource transfer [40,41]. With the extension of stress duration, heavy metal ion accumulation interfered with photosynthesis and root absorption capacity, resulting in a decline in the synthesis and transfer capacity of its endogenous resources [42,43]. This, in turn, actively restricted the transport of resources to the recipient ramets. b. Connective tissue structure and function damage: prolonged HMs and PAHs stress induced membrane lipid peroxidation and cell death in connective tissues [44,45], resulting in the decrease in the transport efficiency of stolon phloem. c. Regulation of hormone signaling and gene expression: Long-term abiotic stress activated jasmonic acid and ethylene signaling pathways while suppressing auxin-mediated resource-sharing genes [46,47]. Supporting these observations, the decline in SPAD values and photosynthetic parameters of donor ramets became less pronounced with prolonged stress, further confirming the gradual weakening of physiological integration effects. Moreover, physiological integration in clonal plants typically involves a cost-benefit trade-off [48,49]. At the initial stage of stress, the integration benefits to recipient ramets significantly outweighed the costs to donor ramets, resulting in overall plant benefit. However, as stress persisted, increased damage to both recipient and donor ramets altered this balance. The donor ramets faced higher integration costs, while recipient ramets received diminished benefits, potentially threatening donor ramet survival. Consequently, donor ramets reduced or even ceased physiological integration to prioritize their own growth and ensure overall plant fitness [50]. This cost-benefit rebalancing was also the important reason for the gradual weakening of physiological integration with the prolongation of stress.

Although the physiological integration intensity under all three stress conditions (Cd, Phe, or their combination) was all weakened with the extension of stress duration, there were differences among these conditions. Environmental heterogeneity serves as a fundamental prerequisite for physiological integration in clonal plants. Studies by You et al., Xing et al., and Wang et al. demonstrated that the higher the heterogeneous contrast of environmental plaques, the more pronounced the physiological integration effect [51,52,53]. In this study, Cd stress exhibited the highest toxicity, followed by the combined Cd and Phe stress, while Phe stress showed the least toxicity. This toxicity gradient can be attributed to two factors: (1) Cd^2+^, as an inorganic ion, is more easily absorbed by plants than Phe [54]; (2) when Cd^2+^ and Phe coexist, they can form cation–π interactions that reduce their bioavailability [55]. Therefore, *Z. japonica* ramets under Cd stress (characterized by high toxicity and thus high contrast) displayed the strongest physiological integration intensity during the early stages (4 d and 8 d). However, the high toxicity also caused the donor ramets to experience greater stress and enter the ‘self-protection state’ more quickly in the later stages, leading to a swift decline in physiological integration intensity. In contrast, *Z. japonica* ramets under Phe stress (characterized by low toxicity and thus low contrast) exhibited the weakest physiological integration intensity in early stages (4 d and 8 d). Nevertheless, the donor ramets experienced less stress and transitioned to the ’self-protection state’ more gradually, resulting in the highest physiological integration strength during late stress periods (16 d and 20 d). This phenomenon revealed the relationship between stress toxicity and temporal patterns of physiological integration: high-toxicity stress initially induces strong physiological integration that rapidly declines, becoming weakest in later stages, while low-toxicity stress shows weak initial integration that declines slowly, becoming strongest in later stages. Due to the limitations of experimental conditions and the current stage of research, the internal mechanism underlying changes in physiological integration intensity, such as cell ultrastructure changes in cells, differential gene expression, and enriched gene pathways, warrants further exploration.

## 4. Materials and Methods

### 4.1. Plant Material

The cultivated *Z. japonica* ramets were used for plant materials, which were provided by the ecological experimental garden at Liaoning University. A pair of connected *Z. japonica* ramets of the same year were transplanted into each pair of pots (one pot is 10 × 10 × 8 cm) within 0.5 kg of dried soil. The basic properties of the soil are shown in Table 2. As the final experimental materials, 70 pairs of *Z. japonica* ramets in total with consistent and healthy growth were selected (size similar, each rooted ramet at least 3 leaves). After a 2 weeks acclimatization period, all pots were moved to the ecological experimental garden for continued growth. The garden’s environmental conditions were maintained at 18.5 ± 2.0 °C to 30.5 ± 2.0 °C, with a relative humidity of 60 ± 5%. The illumination is about 80,000 lux on a clear day and 25,000 lux on a cloudy day. The experiment was conducted in Shenyang (41.8° N, 123.4° E) from August 10 to 30; the natural photoperiod decreased from 14 h (10 August) to 13 h (30 August). Throughout the experiment, the pots were irrigated daily with distilled water to maintain approximately 75% field capacity. Additionally, a weekly application of 0.5 g urea (CAS No.: 57-13-6, AR, 99%, Sinopharm Chemical Reagent Co., Ltd., Shanghai, China) and nothing else was administered as a supplemental nutrient source during the growth period to promote leaf growth.

### 4.2. Experiment Design and Treatment

**Figure 5:**
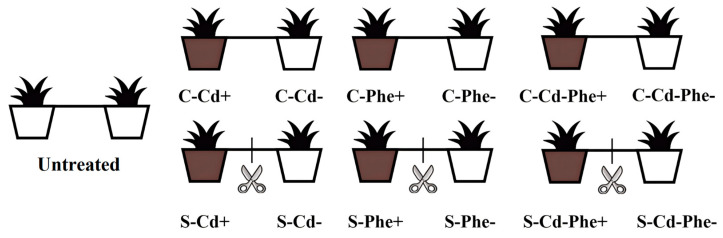
Connected and severed *Z. japonica* ramets under Cd or/and Phe heterogeneous treatments. C: Ramets remained connected (rhizomes intact); S: Ramets were severed (rhizomes cut). Cd+, Phe+, and Cd-Phe+: means the pot was treated with Cd, Phe, and Cd combined with Phe, respectively; Cd-, Phe-, and Cd-Phe-: means the pot was left untreated. Untreated, no contaminants were added to either pot.

Following a 5 weeks acclimatization period in the ecological experimental garden, the 70 pairs of ramets grew well and were randomly divided into two experimental groups: C-group: the two ramets remained connected via stolons; S-group: the two ramets were severed by cutting the stolon. After a 7 d recovery period, according to the pre-experiment and the known tolerance levels of *Z*. *japonica*, one pot from each pair was, respectively, treated with 50 mg·kg^−1^ Cd (Cd+), 100 mg·kg^−1^ Phe (Phe+), and 50 mg·kg^−1^ Cd combined with 100 mg·kg^−1^ Phe (Cd-Phe+), while the paired pot remained untreated (Cd-, Phe-, and Cd-Phe-), establishing a heterogeneous stress condition. CdCl_2_ treatment solution was prepared with deionized water, whereas Phe was first dissolved in 0.1% acetone before dilution with deionized water. No substances were added to the two pots of the untreated group (Figure 5). CdCl_2_ (CAS No.: 10108-64-2, AR, 99%) and Phe (CAS No.: 85-01-8, 99%) were purchased from Shanghai Macklin Biochemical Co., Ltd. (Shanghai, China). Each treatment condition was replicated three times, and the heterogeneous stress treatment was maintained for a duration of 20 d.

### 4.3. Determination of Photosynthetic Parameters and SPAD Values

At 4, 8, 12, 16, and 20 d of heterogeneous stress, P_N_, Cond, C_i_, and Tr were, respectively, measured between 10:00 a.m. and 12:00 p.m. (26–30 °C). Measurements were conducted using a portable open photosynthesis system (LI-6400, Li-Cor, Lincoln, NE, USA), under controlled conditions at a light intensity of 1000 µmol·m^−2^·s^−1^, a CO_2_ concentration of 400 µmol·mol^−1^, and 60% air humidity. Gas exchange was measured from individual leaves, every leaf measurement representing an average of nine readings (three times per leaf, one leaf at the same location per ramet, three ramets per treatment), and WUE was calculated using the formula WUE = Pn/Tr. The SPAD values of leaves were measured using a SPAD-502 (Konica Minolta, Inc., Tokyo, Japan) portable chlorophyll meter. Nine measurements were taken from different zones of each leaf, and the mean value was recorded for analysis.

### 4.4. Definition and Calculation of Physiological Integration Intensity

Physiological integration serves as a crucial adaptive mechanism in clonal plants under heterogeneous stress conditions, effectively mitigating stress impacts on growth, physiological processes, and photosynthetic performance. This characteristic enables distinct responses between plants in physiological integration states compared to non-integrated states. We hypothesize that the magnitude of parameter changes (either increases or decreases) reflects the strength of physiological integration effects, with greater changes indicating more pronounced physiological integration intensity. In this study, we quantified physiological integration intensity by comparing the relative increases in photosynthetic parameters (Pn, Cond, C_i_, Tr, and WUE) and SPAD values between connected (C-Cd+, C-Phe+, and C-Cd-Phe+) and severed (S-Cd+, S-Phe+, and S-Cd-Phe+) treatments. The physiological integration intensity was calculated using the following formula:

Physiological integration intensity = [(C-Cd+) − (S-Cd+)]/(S-Cd+)(1)

= [(C-Phe+) − (S-Phe+)]/(S-Phe+)(2)

= [(C-Cd-Phe+) − (S-Cd-Phe+)]/(S-Cd-Phe+)(3)

This quantitative approach allows for the assessment of integration effects across different stress conditions, providing a standardized method to compare the relative strength of physiological integration under varying environmental challenges.

### 4.5. Statistical Analysis

All experimental data were expressed as mean ± standard deviation (SD). Statistical analyses were performed using SPSS v. 17.0 (SPSS Inc., Chicago, IL, USA). Three-way repeated measure ANOVA was used to examine the effects of duration, connectivity, treatment, and their interactions on photosynthetic indices of *Z. japonica* ramets under Cd or/and heterogeneous stress. A simple effect analysis was performed for the changes in different connectivity of each treatment over the same duration. The method of least significant difference (LSD) was used for post hoc comparison. A probability level of *p* < 0.05 was considered statistically significant. Graphical representations of the data were generated using Origin Pro 2021 (Origin Lab Corporation, Northampton, MA, USA).

## 5. Conclusions

This paper elucidated the physiological integration of photosynthetic parameters and SPAD values of *Z. japonica* ramets in response to Cd or/and Phe heterogeneity stress with different stress durations. In particular, we analyzed the characteristics of physiological integration intensity in relation to stress duration and stress types. Our findings revealed two key characteristics of physiological integration intensity: (1) a progressive decline with prolonged stress exposure and (2) an initial enhancement proportional to stress toxicity during early stress stages, followed by a more rapid attenuation, ultimately resulting in weaker integration strength during later stress phases. These results provide valuable insights into the temporal dynamics of physiological integration under varying stress conditions. Future research should focus on elucidating the underlying mechanisms governing these changes in physiological integration intensity, particularly through comprehensive investigations of cellular ultrastructural modifications and gene expression patterns. Such studies will enhance our understanding of the complex adaptive strategies employed by clonal plants in response to environmental stressors.

## Figures and Tables

**Table 1:** Results of three-way ANOVA for effects of duration (D), connectivity (C), treatment (T), and their interactions (D × C, D × T, C × T, and D × C × T) on photosynthetic parameters and SPAD values in Z. japonica ramets under Cd or/and Phe heterogeneous stress.

		SPAD	P_N_	Cond	C_i_	Tr	WUE
D	F	65.19	6.93	65.33	25.18	11.45	11.85
	P	**	**	**	**	**	**
C	F	85.85	134.36	335.35	2.47	12.06	37.11
	P	**	**	**	ns	**	**
T	F	7683.34	14,670.59	17,876.17	2300.72	9578.60	2862.22
	P	**	**	**	**	**	**
D × C	F	7.17	5.51	247.05	5.20	5.53	3.67
	P	**	**	**	**	**	**
D × T	F	90.56	132.00	128.96	61.00	58.65	39.30
	P	**	**	**	**	**	**
C × T	F	462.32	712.08	1206.04	68.57	509.25	114.05
	P	**	**	**	**	**	**
D × C × T	F	18.64	30.68	94.73	8.70	15.21	6.36
	P	**	**	**	**	**	**

F values and significance levels are given (** *p* < 0.01, ns *p* > 0.05).

**Table 2:** The basic properties of the soil.

pH		Organic Matter Content (g·kg^−1^)		Available Nitrogen (mg·kg^−1^)			Available Phosphorus (mg·kg^−1^)		Available Potassium (mg·kg^−1^)	
7.18		17.60		118			66.5		206	
Ni (mg·kg^−1^)	Cu (mg·kg^−1^)		Pb (mg·kg^−1^)		Zn (mg·kg^−1^)	Mn (mg·kg^−1^)		Cd (mg·kg^−1^)		Cr (mg·kg^−1^)
0.02	2.9		7.9		16.1	3.2		0.04		0.13

## Data Availability

The original contributions presented in this study are included in the article. Further inquiries can be directed to the corresponding author.

## Abbreviations

The following abbreviations are used in this manuscript:

Cd	cadmium
Phe	phenanthrene
HMs	heavy metals
PAHs	heavy metals
P_N_	net photosynthetic rate
Cond	stomatal conductance
C_i_	intercellular CO₂ concentration
Tr	transpiration rate
WUE	water use efficiency
